# Global Analysis of Publicly Available Safety Data for 9,801 Substances Registered under REACH from 2008–2014

**DOI:** 10.14573/altex.1510052

**Published:** 2016-02-11

**Authors:** Thomas Luechtefeld, Alexandra Maertens, Daniel P. Russo, Costanza Rovida, Hao Zhu, Thomas Hartung

**Affiliations:** 1 Center for Alternatives to Animal Testing (CAAT), Johns Hopkins Bloomberg School of Public Health, Environmental Health Sciences, Baltimore, MD, USA; 2 The Rutgers Center for Computational & Integrative Biology, Rutgers University at Camden, NJ, USA; 3 Department of Chemistry, Rutgers University at Camden, NJ, USA; 4 CAAT-Europe, University of Konstanz, Konstanz, Germany

**Keywords:** chemical toxicity, animal testing, database, *in silico*, computational toxicology

## Abstract

The European Chemicals Agency (ECHA) warehouses the largest public dataset of *in vivo* and *in vitro* toxicity tests. In December 2014 this data was converted into a structured, machine readable and searchable database using natural language processing. It contains data for 9,801 unique substances, 3,609 unique study descriptions and 816,048 study documents. This allows exploring toxicological data on a scale far larger than previously possible.

Substance similarity analysis was used to determine clustering of substances for hazards by mapping to PubChem. Similarity was measured using PubChem 2D conformational substructure fingerprints, which were compared via the Tanimoto metric. Following K-Core filtration, the Blondel et al. (2008) module recognition algorithm was used to identify chemical modules showing clusters of substances in use within the chemical universe.

The Global Harmonized System of Classification and Labelling provides a valuable information source for hazard analysis. The most prevalent hazards are H317 “May cause an allergic skin reaction” with 20% and H318 “Causes serious eye damage” with 17% positive substances. Such prevalences obtained for all hazards here are key for the design of integrated testing strategies. The data allowed estimation of animal use.

The database covers about 20% of substances in the high-throughput biological assay database Tox21 (1,737 substances) and has a 917 substance overlap with the Comparative Toxicogenomics Database (~7% of CTD). The biological data available in these datasets combined with ECHA *in vivo* endpoints have enormous modeling potential. A case is made that REACH should systematically open regulatory data for research purposes.

## 1 Introduction

The European REACH legislation (Regulation (EC) 1907/2006)^1^ prescribed the largest collection of chemical toxicity data in history. REACH aims to collect comprehensive safety information for all substances on the European market in volumes of more than 1 ton per year of production or import volume. Basically, it includes three groups of substances, i.e., substances for which so far no registration was necessary on the European level, substances introduced under the Dangerous Substances Directive, since then with somewhat different registration requirements, and all new substances above 1 ton per year since entering into force of the REACH legislation. The legislation is organized by different deadlines, two of which had passed at the time of data analysis. The first required the registration of substances at tonnage levels above 1,000 tons and those with concerns as to carcinogenicity, mutagenicity and reproductive toxicity (CMR) before December 2010 and the second required the registration of substances above 100 tons per year before June 2013; new substances were added to this, but their number is relatively small (Hartung, 2010). For this reason the analysis is clearly biased toward high-production volume substances.

While computational toxicology has recently seen the collection of several large-scale datasets (e.g., US EPA’s ToxCast), the data generated and collected for REACH, owing to its legislative nature, is becoming the largest collection of (eco-)toxicology data relating to *in vitro* and *in vivo* endpoints. However, the REACH dossiers are currently proprietary and any workflows involving the public summary data in REACH depend on the slow and error-prone process of manual extraction. Dossiers can be viewed on the ECHA website^2^; documents are generated by industry via the IUCLID^3^ application.

Here we seek to demonstrate the extent and diversity of the REACH dataset – a dataset that far surpasses most existing datasets used for computational toxicology – and show how an open-access REACH program could allow a profound change in computational toxicology. More detailed analyses were performed for ocular, oral and skin endpoints in other publications (Luechtefeld et al., 2016a–c, this issue).

## 2 Methods

### 2.1 REACH data extraction

Data was downloaded from ECHA using HtmlUnit in an iterative manner in order not to hinder data flow, using an open source Java “Gui-less browser” library (Bowler, 2002). Implementation of ECHA dossier download automation used the functional programming language SCALA (Odersky et al., 2004).

A MongoDB database^4^ was generated from REACH data (Chodorow, 2013). Extracted REACH data is stored as a query-able collection of documents in this Mongo database. The database was generated by automated data extraction from ECHA dossier URLs via the SCALA driver ReactiveMongo (Godbillon, 2015).

Every document is identified by a unique set of three fields:

- *ECNumber:* Substance identifier (“415-890-1”)
- *type:* Study description (e.g., “Exp Key Eye irritation”)
- *num:* disambiguates repeat studies (1, 2, 3,…)

The constructed database, downloaded December 17, 2014, contains 816,048 such documents with 9,801 unique substances (identified by ECNumber) and 3,609 unique study descriptions. Not every substance was associated with information for every study type.

While ECHA disseminated data is a highly structured dataset, much of REACH data contains natural language for quantitative and categorical fields such as: number of animals, Klimisch score, dates, GHS hazards, dose data, response data, etc. These fields were mapped to numeric or categorical values via regular expression recognizing number words and numbers.

To better enable categorization of studies used for animal endpoints, we enriched studies by categorizing into four groups (InVitro, InVivo, ReadAcross or QSAR / PCHEM) mainly through analysis of keywords (i.e., “read across” in the methods data likely represents a ReadAcross study). The QSAR / PCHEM category refers to quantitative structure activity relationship model studies and physicochemical property studies. Due to an overlap in the language used by ECHA to describe these studies, QSAR and PCHEM are grouped together.

When applicable, guideline identifiers were extracted from study data. Thus all the studies matching a given OECD guideline can be easily queried.

ECHA disseminated data is of a highly nested nature: administrative, reference, results, materials and methods data all have many subfields, and some subfields have their own subfields. The root fields for studies may, but do not necessarily, include:

- *ADMIN_DATA:* Klimisch score, data waiving flag, etc.
- *Data source:* References (authors, years, bibliographic sources)
- *Materials & methods:* Study method details
- *Results & discussions:* study result information (e.g., dose-response data)
- Applicant summary & conclusion: result interpretation

Administrative data is associated with most studies in ECHA disseminated data, with the exception typically being a chemical report for classification and labeling. The extracted data may include fields for:

- *Purpose flag:* Four categories for study purpose including: *key study, supporting study, weight of evidence and disregarded study*
- *Data waiving:* Four categories to justify data waiving including: *study not technically feasible, scientifically unjustified, exposure conditions and other justification*.
- *Reliability: 1 (reliable without restrictions), 2 (reliable with restrictions), 3 (not reliable), 4 (not assignable)* and *other* (Klimisch et al., 1997).
- *Study result type:* Study descriptions including: *estimated by calculation, experimental results, (Q)SAR, read-across from supporting substance, read-across based on grouping of substances, no data, experimental study planned* and 2,450 values prefixed by “other”.

Material and methods data associated with studies submitted to ECHA tended to be varied in key fields. Most materials and methods data include:

1. *Materials:* Table of substances used
2. *Organism details:* name, sex, number used, etc.
3. *Guideline:* Information such as OECD guideline
4. *Exposure details:* dose, duration, frequency
5. *Misc:* Many study specific fields

### 2.2 Computational methods

Multiple programming languages, packages and database tools were used in the development of this project. Below we review the use of PubChem and other public databases, the visualization package Gephi and the layout algorithm Force Atlas.

#### PubChem Power User Gateway

PubChem’s Power User Gateway provided data on chemical similarity (including chemical fingerprints), chemical properties including molecular weight, chemical identification information (common names, SMILES, etc.), and bioassay information (Cheng et al., 2014). Bioassay information includes 44,893 assays performed on at least one ECHA chemical and available on PubChem. The data provided by PubChem informed similarity analyses and computational models found in our related publications (Luechtefeld et al., 2016a–c, this issue).

PubChem’s 2D conformational Tanimoto similarity metric^5^ was accessed through the Chemistry Development Kit (Steinbeck et al., 2003). This similarity metric breaks substances into 881 element binary vectors describing the presence or absence of substructures. Similarity between chemical vectors is calculated via Tanimoto distance. Tanimoto distance, the fraction of shared substructures divided by total number of substructures, is a number between 1 (perfectly similar) and 0 (no similarity):

$$T(A,B)=\frac{|A\cap B|}{|A\cup B|}$$

The K-Core algorithm was used to filter out substances with less than 30 neighbors. K-Core, an iterative algorithm, removes substances with the fewest neighbors first until all remaining substances have at least k neighbors. Previous use in protein-protein networks and protein function analysis provide evidence of K-Core’s use in discovering useful network structures (Altaf-Ul-Amine et al., 2003; Alvarez-Hamelin et al., 2005; Wuchty and Almaas, 2005). The parameter 30 was chosen to reduce the network to a manageable number of well connected modules.

#### Module creation

Following K-Core filtration, we used the Blondel (Blondel et al., 2008) module recognition algorithm to identify chemical modules in the K-Core reduced similarity graph. Blondel’s algorithm optimizes Q, a measure of network modularity as evaluated by a function of vertex similarity and module assignment:

$$Q=\frac{1}{2m}\sum_{i,j}\left[A_{ij}-\frac{k_{i}k_{j}}{2m}\right]\;\delta (c_{i},c_{j})$$

In the above formula:

- *A_ij_* is the similarity of chemical *i* and *j,*
- *m* = Σ*_i_*,*_j_ A_ij_* is the total sum of all similarities,
- *k_i_* = Σ*_j_ A_ij_* is the sum of similarities to chemical *i*,
- *c_i_* is the module containing chemical *i*,
- *δ* (*c_i_*, *c_j_*) is 1 if *c_i_* = *c_j_* and 0 otherwise.

*Q* takes on values between −1 and 1. Good modularity, defined by stronger similarity between substances in the same modules versus different modules, is observed for networks with *Q* ≥ 0.3 (Blondel et al., 2008).

#### Gephi

Gephi, a network visualization tool, was used to construct and analyze similarity networks (Bastian et al., 2009). The code for Gephi is openly available^6^ and free to extend or modify.

#### Force layout

The force layout algorithm (Jacomy et al., 2014) was used for generation of chemical similarity networks. The force layout algorithm works on graphs with nodes and edges. Nodes in a graph are connected by edges. The force layout algorithm treats nodes as charged particles that repel each other and edges as physical connections between these particles. The algorithm then positions nodes via a physics simulation.

#### Term Frequency x Inverse Document Frequency (TFIDF)

TFIDF was performed on an 881-dimensional “substructure importance” vector by summing the occurrences of all 881 substructures inside a module (module frequency) and dividing by their frequency in all substances (inverse chemical frequency). We denote this MF_ICF or “Module Frequency Inverse Chemical Frequency”.

$$C(s_{i})=\sum_{\mathrm{chem}}^{\mathrm{chemicals}}\mathrm{contains}(\mathrm{chem},s_{i})$$

Counts occurrence of structure *s_i_* in all substances

$$M_{i}(s_{j})=\sum_{\mathrm{chem}}^{\mathrm{chem}\in M_{i}}\mathrm{contains}(\mathrm{chem},s_{i})$$

Counts occurrence of structure *s_j_* in module *M_i_*

$$S_{i}=\left(\frac{M_{i}(s_{0})}{C(s_{0})},\frac{M_{i}(s_{1})}{C(s_{1})}\ldots \frac{M_{i}(s_{880})}{C(s_{880})}\right)$$

Substructure importance vector for module *i*

$$\mathrm{Similarity}(M_{i},M_{j})=\frac{S_{i}\cdot S_{j}}{|S_{i}\|S_{j}|}$$

*M_i_* and *M_j_* similarity is measured as the cosine of the angle between both substructure importance vectors given here as the vector dot product over vector magnitudes. Module similarity is measured here as the cosine of module substructure importance vectors.

#### Toxicity databases

We aggregated data from multiple toxicologically relevant databases for analysis of biological and chemical structure data and its relationship with studies found in ECHA data.

ToxRefDB was accessed using the web portal given on the Environmental Protection Agency’s (EPA) website^7^. Tox21 data was also accessed through the EPA’s website by downloading^8^. Substances from the Comparative Toxicogenomics Database (CTD) were available for download^9^. Access to the PubChem and ChEMBL libraries was available through web services^10^ (Bolton et al., 2008). Overlaps between databases were found by matching CAS Registry Numbers (CAS RN). The ChEMBL database stores compounds by a unique chemical identifier (ChEMBL ID) and does not contain CAS RN. For this overlay, CAS RN were converted to canonical SMILES and subsequently searched against the ChEMBL library. Because the PubChem and ChEMBL libraries are large and accessed via web services, the overlap between these databases was taken as reported by The European Bioinformatics Institute^11^.

The results of assays found in PubChem and ChEMBL for high production volume compounds were aggregated using the PubChem Power User Gateway and ChEMBL API. The response of a compound in a given assay was recorded independent of the experimental outcome (e.g., active, inactive, inconclusive, etc.). The assays within CTD were available using the batch query portal within the site^12^. Each chemical-gene interaction for a queried compound was recorded as a response.

## 3 Results

### 3.1 Extracted data overview

Efforts to determine chemical hazards such as eye irritation, skin sensitization and other health hazards have resulted in the accumulation of large amounts of privately held toxicity data. REACH legislation has resulted in the most extensive effort to systematically collect such data and outlined the necessary additional chemical testing that must be done. The constructed database, downloaded December 17, 2014, contains 816,048 such documents with 9,801 unique substances (identified by ECNumber) and 3,609 unique study descriptions.

**Fig. 1:**
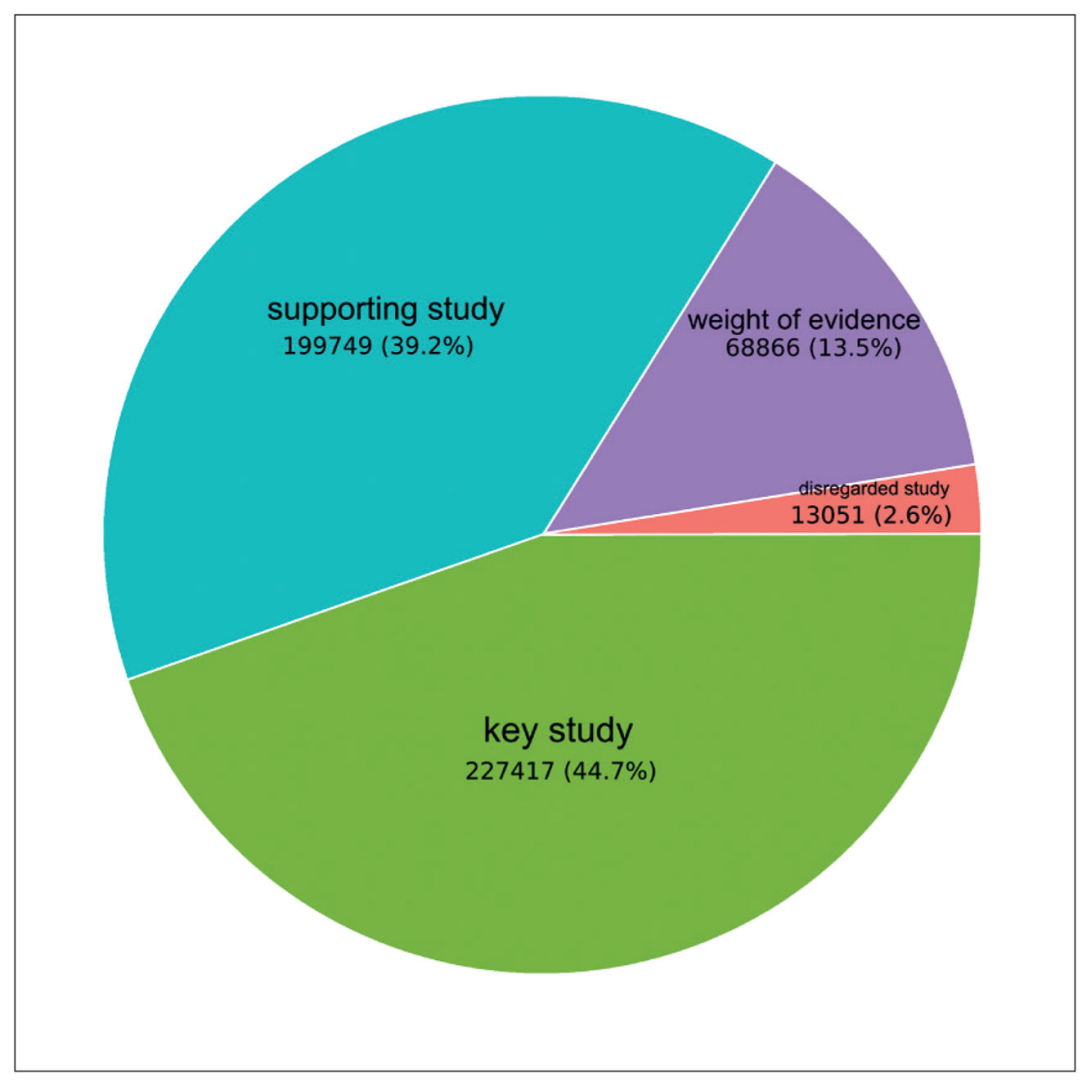
Prevalence of purpose flags Prevalence of the four purpose flags (disregarded study, key study, weight of evidence) over an extraction of 509,083 studies with purpose flags in REACH registrations 2008–2014.

Out of the 509,083 studies with a purpose flag in the extracted data, 13.5% (68,866) have the purpose flag “weight of evidence”, 2.5% (13,051) “disregarded study”, 44.7% (227,417) “key study”, and 39.2% (199,749) have the purpose flag “supporting study” (Fig. 1). Purpose flags can be useful for defining the breadth of database queries; some analyses may only have interest in study results directly used for classification and labeling and should refine their searches to studies with purpose flag “key study”.

**Fig. 2:**
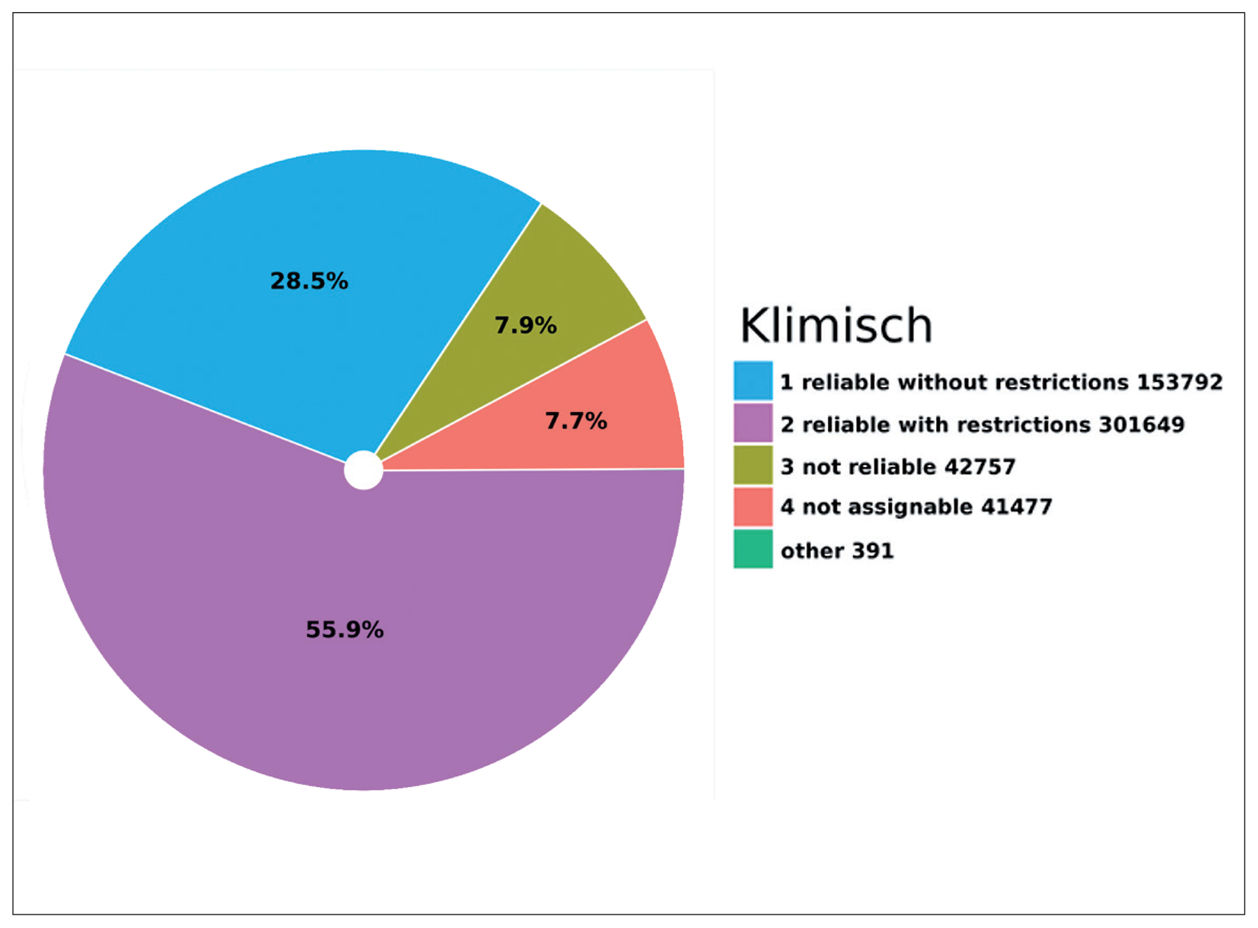
Klimisch score pie chart Prevalence of different Klimisch values over 539,675 studies with assignable Klimisch values in REACH registrations 2008–2014.

Klimisch reliability scores (Klimisch et al., 1997), which are defined by dossier registrants, can also be used to refine searches or even to compare results across reliability levels. Out of 539,675 studies with an assigned reliability score, 30.9% (153,792) have a reliability score of “1 (reliable without restriction)”, 60.5% (301,649) “2 (reliable with restrictions)”, 8.6% (42,757) “3 (not reliable)”, and 8.3% (41,477) have a reliability score of “4 (not assignable)” (Fig. 2).

### 3.2 PubChem chemical similarity

**Fig. 3:**
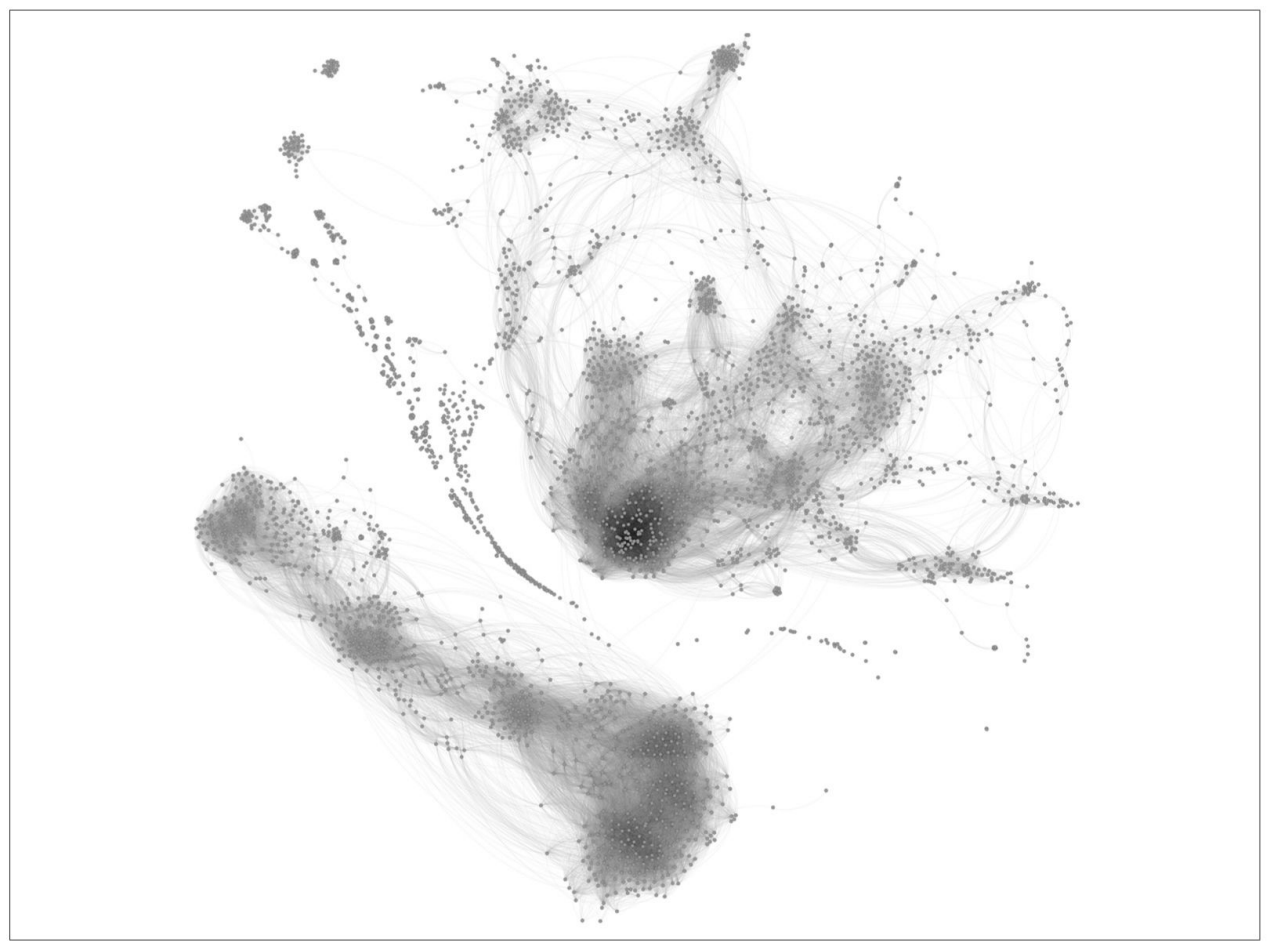
Chemical similarity for 3,122 substances mapped from ECHA dossiers to PubChem Minimum similarity of 0.6. Substances without neighbors are filtered out. Gephi algorithm “Force Layout 2” used for layout.

Mapping substances from REACH to PubChem enables the analysis of chemical similarity via PubChem 2D conformational substructure fingerprints (Jaworska and Nikolova-Jeliazkova, 2007; Cheng et al., 2014; Steinbeck et al., 2003). Substructure fingerprints can be used in combination with the Tanimoto distance (number of shared substructures divided by total number of substructures) to build the chemical similarity map in Figure 3. We employed the 2D conformational fingerprint, which treats each fragment as 1 or 0 depending on its presence in a substance. Similarity is calculated as the number of shared fragments divided by the total number of fragments in both molecules. Although other similarity measures exist for binary vectors, we chose Tanimoto for its simplicity (Lourenço et al., 2004). More advanced similarity measures can be expected to perform more strongly than the baseline-setting approach used here.

Large chemical similarity graphs allow both visualization of the global chemical diversity of a dataset and suggest different chemical classes within in the data. In construction of the chemical similarity network, filtering was performed for visualization and identification of network modules. Edges between substances with similarity less than 0.65 were discarded.

**Fig. 4:**
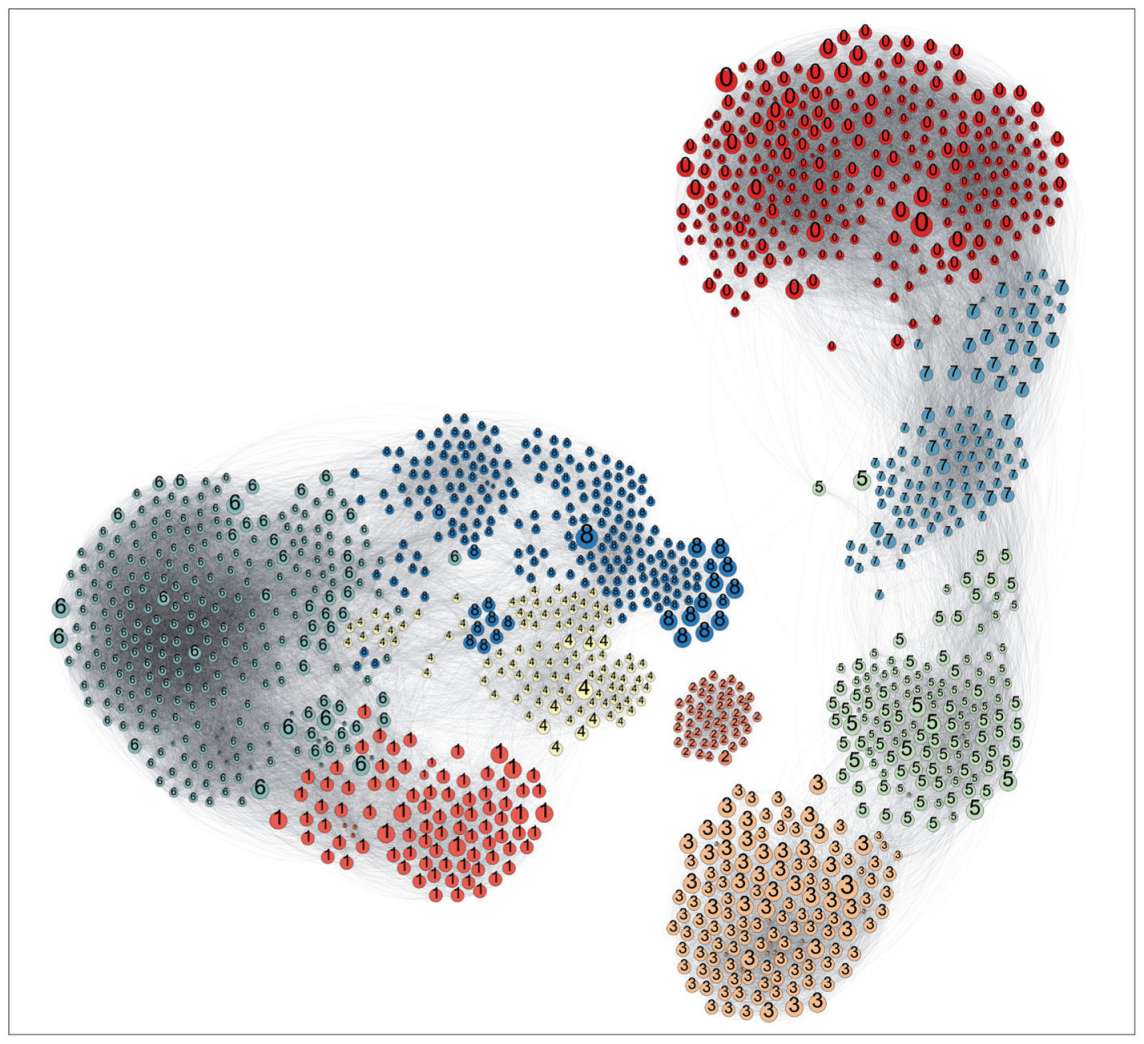
Filtering of chemical similarity graph via K-Core Chemical coloring via module membership (determined by Blondel et al. (2008) algorithm) to the nine global modules numbered 0–8.

Edge filtration and K-Core chemical filtration reduce 3,122 original substances (mapped from REACH to PubChem) to 1,383 and number of edges from 84,993 to 69,041. Preservation of one third of the original population demonstrates the well-connectedness of the entire chemical similarity network. Figure 4 shows the resulting filtered chemical similarity map with substances colored by modularity.

The REACH extraction network modularity *Q* value of 0.688 demonstrates strong modularity. Supporting evidence of strong modularity comes from visual inspection of the resulting map (with 9 modules given unique colors). Three large disconnected modules can be seen divided into visually reasonable neighborhoods. Edge similarity is visualized via transparency, with opaque edges of higher similarity and translucent edges of low similarity; tightly connected modules are observed to display dark, strongly weighted edges.

#### 3.2.1 Gephi force layout visualization

Layout and visualization relies on the force layout algorithm implemented within an open source Java network visualization software called Gephi (Bastian et al., 2009). While technical details are beyond the scope of this paper, ForceAtlas distributes edges and nodes by simulating a physical system where nodes repulse each other (like charged particles) and edges attract their attached nodes (like springs) (Jacomy et al., 2014).

**Fig. 5:**
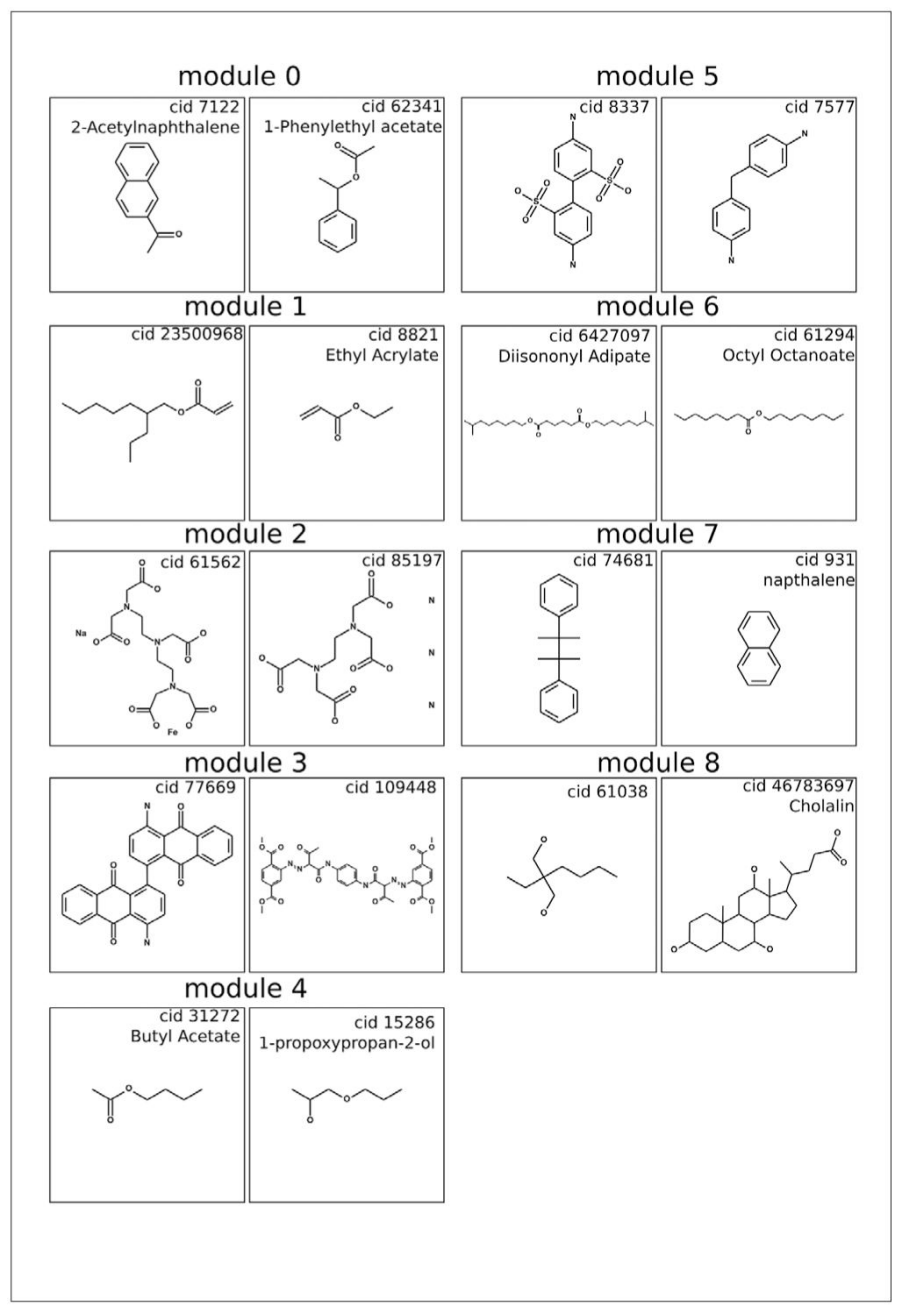
Chemical examples from each module in Figure 4

Substances are colored by their module number in Figure 4, and several example substances from each module are shown in Figure 5. While the Blondel et al. modularity algorithm provides a strong determination of global modules, it is interesting to consider the intra-module cohesiveness. Module cohesiveness, as measured by comparing similarity between substances in a module to substances outside a module, is the basis for Blondel algorithm module identification (Blondel et al., 2008). For example, visual inspection shows that module 8 is not a very cohesive module and could be broken up into several sub modules, and the chemical examples chosen from module 8 are selected from disparate submodules and do not appear strongly related. Module 2 showed extremely high intra-connectivity and structurally very similar substances – this likely reflects a class for which using a SAR approach could be fruitful.

#### 3.2.2 Module analysis

The super modules discovered via the Blondel algorithm have varying inter- and intra-connectedness. For instance, Module 2, Modules (1, 4, 6, 8) and Modules (0, 7, 5, 3) form 3 super modules with high degrees of interconnectivity.

To attempt to investigate and quantify this connectivity we borrowed the “Term Frequency x Inverse Document Frequency (TFIDF)” approach from document retrieval literature (Salton et al., 1975). TFIDF is often used in text-mining to assess the “importance” of a word by calculating its frequency in a given document in comparison to its typical appearance in the broader corpus, e.g., for a word to have a high value it must appear frequently in a document, but infrequently in other documents. We adapted this approach for chemical substructures to examine which substructures were the most informative for each module. Table 1 gives the highest ranking 10 substructures in each module.

Table 2 gives the similarity between each module measured in this way. The results help to confirm the validity of the TFIDF approach. Modules that appear visually related (Fig. 4) also have high quantitative similarity. Example substances were chosen from each module to help visualize the module constituency. The examples are given in Figure 5 and help to inform module characterization.

Three super modules, modules (1, 4, 6, 8), modules (0, 7, 5, 3), and module 2, can easily be visualized. The two bigger super modules, modules (1, 4, 6, 8) and modules (0, 7, 5, 3), differ mainly in the frequency of straight-chain and cyclic alkanes or aromatic rings, respectively. In the first super module, modules (1, 4, 6, 8), modules 1 and 6 are both long and short chain esters differing only in the degree of saturation of their alkyl chains, explaining the high amount of similarity between the modules. Module 8 showed highly-cyclic structures of varying ring size and showed intermodular similarity with module 6 due to the O-C-R substructures contained in the cyclic alcohols and the esters. Another super module, module 2, is based on glycine derivatives that share little similarity with all other modules. The slight overlap with module 4, a module with ester and ether derivatives, comes from the shared O=C-O-R moiety in both groups. The other large super module, modules (0, 7, 5, 3), also shows some obvious feature overlaps. Module 0 is characterized by a high frequency of alcohol derivatives and esters, and showed the highest intermodular similarity with module 7, a module showing a high frequency of thiols. The similarity is most likely owing to the frequency of aromatic cyclic structures with a lone substitution in both groups. Module 3 (quinone and glycine derivatives) and module 5 (dianilines) shared high intermodularity due to the shared aniline backbone.

### 3.3 OECD guideline usage

ECHA studies designate OECD guideline numbers when appropriate. These numbers improve analysis because studies sharing the same OECD guideline can be expected to have similar data formats (materials and methods, results, etc.). Table 3 shows the top 3 OECD guidelines for each enriched category (InVivo, InVitro, QSAR / PCHEM, Read Across). It should be noted that since OECD guidelines are given by ECHA in natural language and were extracted via regular expression recognition, it is possible that some guidelines were extracted imperfectly.

REACH requirements for *in vitro* skin corrosion, skin irritation, eye irritation, and bacterial gene mutation are described in Annex VII., i.e., for all tonnage bands (Aulmann and Pechacek, 2014; European Commission, 2006). As these endpoints are required for large numbers of substances, they should have a high frequency in the extracted data. Given this constraint, it is surprising that none of the OECD skin sensitization guidelines appear near the top in Table 3. Automatic curation indicates that out of the 9,801 extracted substances 5,551 were missing explicit *in vivo* key experimental skin sensitization studies, possibly due to data waiving or being substituted by read-across methods. Manual inspection of six online ECHA dossiers of substances missing key experimental *in vivo* sensitization testing agreed with the automatically extracted results and identified the following:

- *919-583-6:* No key skin sensitization study given
- *206-768-5:* Data waiving (other justification)
- *920-191-2:* Read-Across GPMT (category approach)
- *923-592-0:* Read-Across Mouse LLNA (substitute 269-646-0)
- *482-090-5:* No key skin sensitization study given
- *273-748-0:* Read-Across Mouse LLNA (substitute 273-733-9)

Analysis of the substances missing a key skin sensitization study indicated that out of 637 skin sensitization studies with data waiving, 360 are labeled as “other justification”, 255 are classified as “study scientifically unjustified”, and 148 as “study technically not feasible”. Examination of study result types associated with substances without a skin sensitization study indicate 2,735 read-across from supporting substance, 2,156 read-across based on grouping of substances, 2,144 experimental result, 157 “estimated by calculation”, and 128 (Q)SAR. This data indicates that read-across from a supporting substance is a more prevalent study type than read-across from categorization for substances lacking a key experimental skin sensitization study.

TG 401: Acute Oral Toxicity (OECD, 1987) is the third most prevalent *in vivo* OECD TG in the extracted database. It is also the second most prevalent guideline in the read-across category. REACH stipulates in Annex VII that acute toxicity must be evaluated for all tonnage bands, thus corroborating the extraction’s high prevalence (Aulmann and Pechacek, 2014). Overlaps in *in vitro* and read-across OECD guidelines indicate potentially rich datasets for the evaluation of read-across approaches.

OECD guideline data is used extensively in other publications in this issue evaluating ocular, skin and oral toxicity in more depth (Luechtefeld et al., 2016a–c, this issue).

### 3.4 Hazard distribution

**Fig.6:**
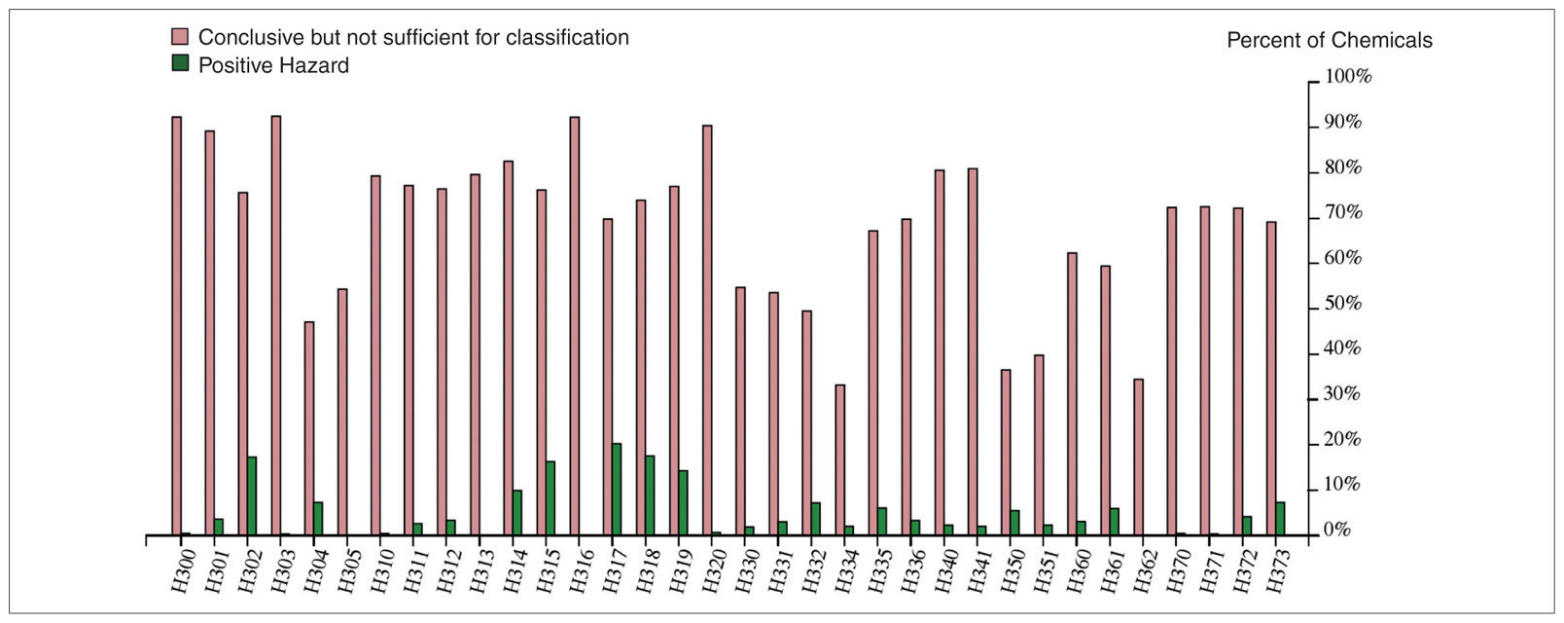
Frequency of different health hazards in extracted dataset of REACH registrations 2008–2014 Hazard definitions given in Table 4 (Hazard values extracted for 6,186 substances). Green bars designate the frequency of chemicals labeled with the given hazard, red bars designate the frequency of chemicals not labeled with given hazard.

ECHA dossier submissions contain classification and labeling data that can be mapped to hazard definitions given by the Globally Harmonized System of Classification and Labelling. Figure 6 identifies label frequency as reported in extracted ECHA dossiers. Extracted GHS values exist for 6,186 REACH substances; incomplete GHS extractions are due to the limitations in text analysis and occasional inconsistencies in data format.

The most abundant hazard is H317 “May cause an allergic skin reaction” with 1,255 (20%) labelled substances, 4,317 (70%) substances with “conclusive but not sufficient data for classification” (which designates that data are available indicating no need for classification), 428 (6%) substances recorded as “data lacking”, 26 (0.4%) substances recorded as “inconclusive” and 160 (2.5%) substances for which data extraction failed. The high frequency of this hazard, the relatively well-established Adverse Outcome Pathway (AOP), as well as the relative ease of using *in vitro* tests for various steps of the pathway make it an ideal test case for further research into Integrated Testing Strategies (Hartung et al., 2013). For a more detailed analysis of the skin sensitization data see Luechtefeld et al. (2016c, this issue).

H318 “Causes serious eye damage” is the second most frequent endpoint with 1,087 (17%) positive substances, 4,574 (74%) “conclusive but not sufficient”, 352 (5.7%) “data lacking”, 14 (0.02%) “inconclusive” and 159 (2.5%), for which data extraction failed. We examine ocular toxicity in more detail in Luechtefeld et al. (2016b, this issue).

The information on hazard frequencies in Table 4 can be used as estimates for hazard prevalence to anchor testing strategies (Hoffmann and Hartung, 2005).

### 3.5 Animal use

The number of animals used in REACH data sources can be extracted simply from Materials and Methods data. In a given study the number of animals used is given in natural text, e.g., “5 males and females”. We wrote heuristics for extracting animal counts from these natural language descriptions. Additionally, due to lack of reference identifiers, the same reference may be counted multiple times when it is used for different ECHA studies, thus inflating the estimates.

**Fig. 7:**
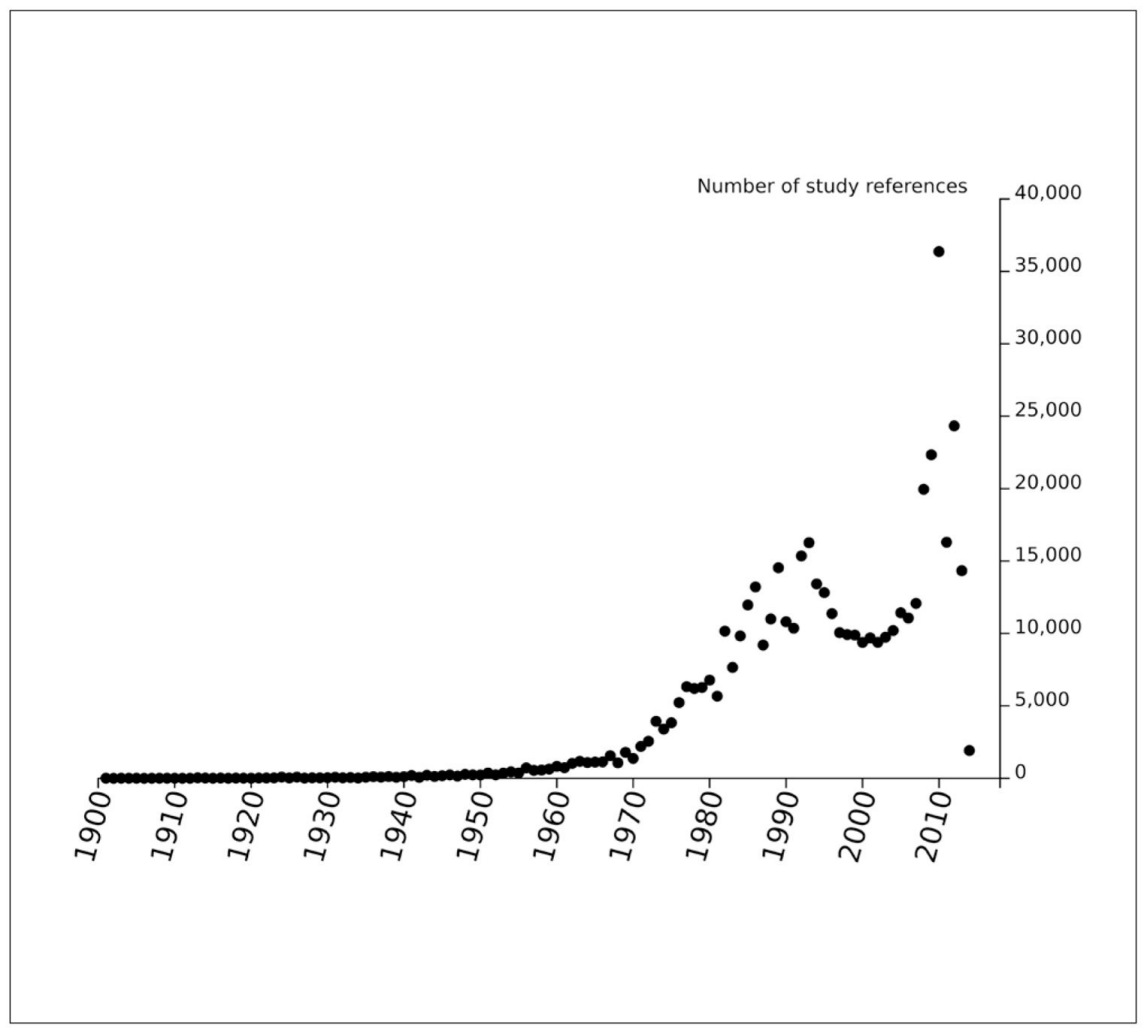
Number of sources from each year Possible double counting due to absence of reference identifiers in ECHA dossiers.

**Fig. 8:**
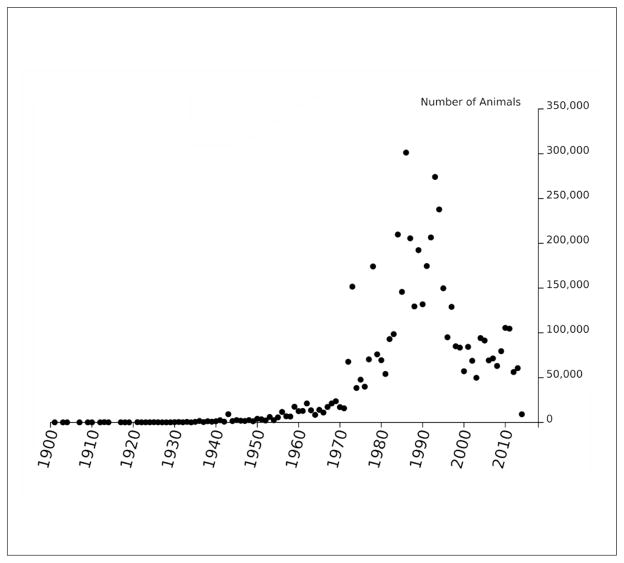
Number of animals used in data sources from referenced year in REACH registrations 2008–2014 Possible double counting due to missing reference identifiers in ECHA dossiers.

We can evaluate use of animals in reference studies over time by first assessing the distribution of study start dates (Fig. 7) and then finding the distribution of number of animals used in each year (Fig. 8). We used simple heuristics to estimate animal counts from natural language. When comparing Figure 7 and 8 it appears that the number of animals used per reference was lower in the late 2000s relative to the 1990s.

### 3.6 Data overlap

To determine the relevance of ECHA extracted data in the context of current toxicological databases, the 9,801 extracted REACH compounds were searched against three well-known toxicity datasets: Toxicity Reference Database (ToxRefDB), Toxicity Testing in the 21^st^ Century (Tox21) and Comparative Toxicogenomics Database (CTD).

ToxRefDB is a collection of 30 years of animal toxicity testing data in the US Environmental Protection Agency (US EPA) and contains 474 compounds (Martin et al., 2009).

Tox21 is a collaborative screening effort among EPA, the National Institute of Environmental Health Science (NIEHS), National Toxicology Program (NTP), the National Center for Advancing Translational Sciences (NCATS), and the Food and Drug Administration (FDA) (Tice et al., 2013): Phase I of Tox21 investigates approximately 2,800 compounds in over 75 bioassays. Phase II expanded the chemical library to over 10,000 and seeks to test these compounds in approximately 40 assays over the coming years (Attene-Ramos et al., 2013). This target chemical library mainly consists of compounds of environmental interest (e.g., high production volume compounds, pesticides, drugs, etc.).

The CTD consists of 13,446 compounds with toxicogenomics data (e.g., drug molecules). This public database aims to explore how environmental exposures impact human health via manually curated chemical-gene, chemical-protein, chemical-disease and gene-disease interactions.

REACH compounds have the largest overlap (1,737 compounds) with Tox21 compounds, possibly reflecting the similar goals of Tox21 and REACH (Tab. 5). The overlap between REACH and CTD is much lower. The extracted REACH substances cover 11% of Toxcast, 20% of Tox21 and 7% of CTD. The biological data available in these datasets combined with *in vivo* endpoints extractable from REACH represent a strong modeling potential.

PubChem, a large chemical database hosted by the National Center for Biotechnology Information (NCBI) and the National Institutes of Health (NIH) (Cheng et al., 2014), currently contains 68 million compounds tested in over 1 million bioassays, including massive amounts of toxicity data. It is not surprising that 4,955 of the REACH substances are found here. ChEMBL, established by the European Bioinformatics Institute, is part of the European Molecular Biology Laboratory (EMBL). ChEMBL is a chemical-bioassay database manually curated from peer-reviewed publications consisting mostly of drug-like compounds (Gaulton et al., 2011), but 2,080 of the REACH chemicals are also represented here. Both repositories are thus very rich for further analysis.

## 4 Discussion

Massive amounts of toxicity data have been generated in the past decade and various data repositories have been developed to share data with research communities. REACH is the largest of these efforts with expected multi-billion Euros of testing costs (Hartung and Rovida, 2009; Rovida and Hartung, 2009), but so far its full potential has not been realized. A searchable repository of the publically available REACH data represents an enormous resource for toxicology, particularly computational approaches requiring large datasets.

REACH data can be used to inform risk assessments, develop computational models, develop and evaluate test strategies, and improve / store toxicological knowledge on a per study basis. The extracted data is far from perfect as the non-standardized presentation of data in many narrative fields is prone to errors when extracted automatically with search engines. While the primary objective of REACH submissions is not data extraction and mining, this publication and others in this issue (Luechtefeld et al., 2016a–c) demonstrate the potential value of ECHA reports submitted for REACH. Further curation, as well with data from registrations occurring post December 2014, would be extremely helpful.

Ultimately, reduction of animal testing will depend in a large part on the development of *in silico* models such as QSAR (Zvinavashe et al., 2009; Patlewicz et al., 2013, 2015). Improvement of computational models relies on accessibility of training and testing data. The open data nature of Tox21, ToxRefDB, PubChem, CTD and ChEMBL promotes numerous publications and development of ever improving statistical and expert models. Overlaps of REACH with existing databases given in Table 5 further demonstrate the value of the extracted data: ToxRefDB (a commonly used animal testing database) covers only 474 substances with multiple animal endpoints while the extraction in this publication covers over 9800.

## 5 Conclusion

The extracted ECHA dataset first of all allows us to better understand the landscape of substances for a given hazard: Which parts of the chemical universe are associated with a given hazard? How concordant and reproducible are different methods? With the limited information of the New Chemicals Database (NCD) of the EU (which is not publicly available), it has previously been shown how much useful information can be extracted from such databases using the example of skin irritation (Hoffmann et al., 2005). Our parallel articles in this *ALTEX* issue address the most prevalent human hazards, i.e., oral toxicity, skin sensitization and eye irritation.

One goal of this publication is to underscore the importance of structuring data in a machine-readable format – while REACH in many ways has a workable ontology for classifying endpoints, the toxicological value of REACH data could be realized by using formal data structures for results extracted from the main guideline-compliant studies, especially for the key hazards of eye irritation and skin sensitization, which easily lend themselves to this approach. Eventually the development of ontologies (e.g., OpenTox, ToxML) to classify studies by type and study results and outcomes for more complicated endpoints, such as developmental toxicity, will greatly aid the ability of toxicologists to assemble large datasets.

Furthermore, it is our hope that our arguments and referenced articles will motivate the systematic and more comprehensive publication of REACH data to the general public. An open REACH platform would allow third parties to investigate concepts such as OECD TG use and quality assessment, testing redundancies, and hazard distributions, and could automate many research tasks.

As we have demonstrated, REACH would provide computational toxicology with an unparalleled dataset for QSAR development, *in vitro* to *in vivo* extrapolation, and computational toxicology approaches. Making REACH open and available to the community should be a priority for both scientists and legislators.

## Figures and Tables

**Tab. 1:** Characteristic substructures for each module ( Fig. 4) as determined by modular frequency x inverse chemical frequency (MF_ICF) Orange = Hierarchic Element Counts. Grey = Rings in a canonic extended smallest set of smallest rings (ESSR) ring set. Green = Simple atom nearest neighbors. Blue = SMARTS patterns. High MF_ICF numbers indicate stronger relationships between the given module and substructure.

FP	MF_ICF	FP	MF_ICF	FP	MF_ICF
**Module 0**		**Module 1**		**Module 2**	
O=C-O-C:C	0.92	C-O-C-C=C	0.25	O=C-C-N-C	0.49
OC1C(O)CCCC1	0.88	C=C-C-O-C	0.25	≥1 Fe	0.31
Oc1c(O)cccc1	0.87	O=C-C=C-[#1]	0.18	O=C-C-N	0.3
Cc1c(O)cccc1	0.84	O=C-C=C	0.16	≥1 Cu	0.26
O-C:C-O-[#1]	0.83	O-C-C=C	0.15	N-C-C-N-C	0.23
O-C:C-O	0.82	C-C-O-C-C	0.13	O-C-C-N-C	0.22
O=C-C:C-O	0.8	O(~C)(~C)	0.11	N-C-C-N	0.2
O-C:C-O-C	0.8	≥1 Sn	0.11	O-C-C-N	0.16
C-C:C-O-[#1]	0.77	C(-C)(-O)(=O)	0.1	≥2 Na	0.16
Cc1ccc(O)cc1	0.75	C(-O)(=O)	0.09	≥8 O	0.15
**Module 3**		**Module 4**		**Module 5**	
Nc1c(Cl)cccc1	0.87	O-C-C=O	0.33	S=C-N-[#1]	0.33
NC1C(Cl)CCCC1	0.87	O=C-C-O	0.33	C-S-C:C	0.32
O=C-C-C-C-C(N)-C	0.86	O=C-C-C-O	0.24	C(~N)(:C)(:C)	0.31
C-C=N-N-C	0.85	O-C-C-C=O	0.24	Cc1ccc(N)cc1	0.31
N-N-C:C	0.84	≥1 Zr	0.22	N-C-C-C:C	0.31
N(~C)(~H)(~N)	0.84	O=C-C-O-C	0.18	N-C-C:C-C	0.31
C=N-N-C	0.83	O-C-C-O-[#1]	0.18	N-C-C:C	0.3
N(~H)(~N)	0.79	≥1 Pb	0.17	CC1CCC(N)CC1	0.3
N-N-C-C	0.79	O=C-C-C-C-O	0.17	C(-C)(-N)(=C)	0.3
≥5 unsaturated non-aromatic carbon-only ring size 6	0.71	O-C-C-O	0.16	N-C:C:C-C	0.29
**Module 6**		**Module 7**		**Module 8**	
O=C-C-C-C-C-C	0.33	Cc1ccc(S)cc1	0.29	CC1CC(O)CC1	0.97
≥1 Sn	0.32	CC1CCC(S)CC1	0.29	CC1C(O)CCC1	0.97
O=C-C-C-C-C	0.32	N-S-C:C	0.28	≥3 saturated or aromatic carbon-only ring size 6	0.93
O-O	0.31	C(~C)(~H)(~P)	0.25	≥2 saturated or aromatic carbon-only ring size 6	0.75
O=C-C-C-C-C-C-C	0.29	Cc1ccc(C)cc1	0.24	≥2 saturated or aromatic carbon-only ring size 5	0.74
O-C-C-C-C-C-C-C	0.29	C(-C)(-Cl)(=O)	0.2	CC1C(C)CCC1	0.71
O=C-C-C-C	0.29	N-S	0.18	≥1 saturated or aromatic carbon-only ring size 5	0.71
C(-C)(-O)(=O)	0.28	C(-C)(-H)(=O)	0.18	CC1CC(C)CC1	0.69
O-C-C-C-C-C-C	0.27	C-P	0.17	CC1CC(O)CCC1	0.53
O-C-C-C-C-C	0.27	S-C:C-C	0.14	≥1 saturated or aromatic carbon-only ring size 6	0.48

**Tab. 2:** Intermodular similarity as determined by cosine of angle between module substructure importance vectors Substructure importance vectors are determined via analog to TFIDF, where a module’s importance for a given substructure is given by its frequency within the module multiplied by the inverse of its frequency in all substances. Green cells show the greatest similarity for the module in each row. These similarities fit well with visual inspection of Figure 4.

Module	0	1	2	3	4	5	6	7	8
**0**	1.00	0.26	0.02	0.24	0.18	0.19	0.26	0.36	0.18
**1**	0.26	1.00	0.06	0.09	0.23	0.08	0.43	0.16	0.15
**2**	0.02	0.06	1.00	0.06	0.10	0.05	0.10	0.04	0.02
**3**	0.24	0.09	0.06	1.00	0.04	0.53	0.11	0.32	0.05
**4**	0.18	0.23	0.10	0.04	1.00	0.02	0.43	0.04	0.22
**5**	0.19	0.08	0.05	0.53	0.02	1.00	0.13	0.40	0.04
**6**	0.26	0.43	0.10	0.11	0.43	0.13	1.00	0.20	0.30
**7**	0.36	0.16	0.04	0.32	0.04	0.40	0.20	1.00	0.09
**8**	0.18	0.15	0.02	0.05	0.22	0.04	0.30	0.09	1.00

**Tab. 3:** Top 3 OECD TG counts by category in REACH registrations 2008–2014 Counts give total number of studies following the given OECD TG.

Category	OECD TG	Count	Description

InVitro	471	6044	Bacterial Reverse Mutation Test (OECD, 1997)
	431	3576	*in vitro* Skin Corrosion: Human Skin Model Test (OECD, 2014)
	435	3287	*in vitro* Membrane Barrier Test Method for Skin Corrosion (OECD, 2006)

QSAR/PCHEM	105	2920	Water Solubility (OECD, 1995b)
	109	2420	Density of liquids and solids (OECD, 2012a)
	102	2322	Melting Point/Range (OECD, 1995a)

InVivo	404	8548	Acute Dermal Irritation/Corrosion (OECD, 2002)
	405	8142	Acute Eye Irritation/Corrosion (Draize) (OECD, 2012b)
	401	7852	Acute Oral Toxicity (OECD, 1987)

ReadAcross	471	3896	Bacterial Reverse Mutation Test (OECD, 1997)
	401	2747	Acute Oral Toxicity (OECD, 1987)
	201	2679	Cyanobacteria Growth Inhibition Test (OECD, 2011)

**Tab. 4:** Hazard counts for extracted GHS hazards in REACH registrations 2008–2014 6,186 substances had extractable classification and labeling data in ECHA dossiers.

Description	Hazard	Labeled substances	Conclusive but not sufficient for classification	Data lacking	Inconclusive	Failed extraction
Unstable explosive	H200	6 (0.1%)	5507 (89%)	492 (8%)	22 (0.4%)	159 (2.6%)
Explosive; mass explosion hazard	H201	14 (0.2%)	5499 (88.9%)	492 (8%)	22 (0.4%)	159 (2.6%)
Fire or projection hazard	H204	16 (0.3%)	5497 (88.9%)	492 (8%)	22 (0.4%)	159 (2.6%)
Extremely flammable gas	H220	41 (0.7%)	4841 (78.3%)	1122 (18.1%)	23 (0.4%)	159 (2.6%)
Flammable gas	H221	2 (0%)	4880 (78.9%)	1122 (18.1%)	23 (0.4%)	159 (2.6%)
Extremely flammable liquid and vapour	H224	90 (1.5%)	5047 (81.6%)	877 (14.2%)	13 (0.2%)	159 (2.6%)
Highly flammable liquid and vapour	H225	268 (4.3%)	4869 (78.7%)	877 (14.2%)	13 (0.2%)	159 (2.6%)
Flammable liquid and vapour	H226	401 (6.5%)	4741 (76.6%)	872 (14.1%)	13 (0.2%)	159 (2.6%)
Combustible liquid	H227	2 (0%)	5136 (83%)	876 (14.2%)	13 (0.2%)	159 (2.6%)
Flammable solid	H228	68 (1.1%)	5277 (85.3%)	659 (10.7%)	23 (0.4%)	159 (2.6%)
May react explosively even in the absence of air	H230	2 (0%)	4880 (78.9%)	1122 (18.1%)	23 (0.4%)	159 (2.6%)
Heating may cause a fire or explosion	H241	4 (0.1%)	4879 (78.9%)	1126 (18.2%)	17 (0.3%)	160 (2.6%)
Heating may cause a fire	H242	55 (0.9%)	4831 (78.1%)	1123 (18.2%)	17 (0.3%)	160 (2.6%)
Catches fire spontaneously if exposed to air	H250	13 (0.2%)	5077 (82.1%)	918 (14.8%)	19 (0.3%)	159 (2.6%)
Self-heating; may catch fire	H251	13 (0.2%)	4886 (79%)	1109 (17.9%)	19 (0.3%)	159 (2.6%)
Self-heating in large quantities; may catch fire	H252	8 (0.1%)	4892 (79.1%)	1108 (17.9%)	19 (0.3%)	159 (2.6%)
In contact with water releases flammable gases which may ignite spontaneously	H260	15 (0.2%)	5076 (82.1%)	914 (14.8%)	22 (0.4%)	159 (2.6%)
In contact with water releases flammable gas	H261	8 (0.1%)	5083 (82.2%)	914 (14.8%)	22 (0.4%)	159 (2.6%)
May cause or intensify fire; oxidizer	H270	3 (0%)	4862 (78.6%)	1139 (18.4%)	23 (0.4%)	159 (2.6%)
May cause fire or explosion; strong oxidizer	H271	15 (0.2%)	5262 (85.1%)	732 (11.8%)	18 (0.3%)	159 (2.6%)
May intensify fire; oxidizer	H272	41 (0.7%)	5236 (84.6%)	732 (11.8%)	18 (0.3%)	159 (2.6%)
Contains gas under pressure; may explode if heated	H280	60 (1%)	4784 (77.3%)	1161 (18.8%)	22 (0.4%)	159 (2.6%)
Contains refrigerated gas; may cause cryogenic burns or injury	H281	1 (0%)	4843 (78.3%)	1161 (18.8%)	22 (0.4%)	159 (2.6%)
May be corrosive to metals	H290	125 (2%)	3722 (60.2%)	2155 (34.8%)	25 (0.4%)	159 (2.6%)
Fatal if swallowed	H300	33 (0.5%)	5709 (92.3%)	273 (4.4%)	12 (0.2%)	159 (2.6%)
Toxic if swallowed	H301	225 (3.6%)	5518 (89.2%)	272 (4.4%)	12 (0.2%)	159 (2.6%)
Harmful if swallowed	H302	1072 (17.3%)	4677 (75.6%)	266 (4.3%)	12 (0.2%)	159 (2.6%)
May be harmful if swallowed	H303	23 (0.4%)	5720 (92.5%)	272 (4.4%)	12 (0.2%)	159 (2.6%)
May be fatal if swallowed and enters airways	H304	453 (7.3%)	2913 (47.1%)	2626 (42.5%)	35 (0.6%)	159 (2.6%)
May be harmful if swallowed and enters airways	H305	3 (0%)	3361 (54.3%)	2628 (42.5%)	35 (0.6%)	159 (2.6%)
Fatal in contact with skin	H310	30 (0.5%)	4905 (79.3%)	1074 (17.4%)	18 (0.3%)	159 (2.6%)
Toxic in contact with skin	H311	164 (2.7%)	4774 (77.2%)	1071 (17.3%)	18 (0.3%)	159 (2.6%)
Harmful in contact with skin	H312	209 (3.4%)	4728 (76.4%)	1072 (17.3%)	18 (0.3%)	159 (2.6%)
May be harmful in contact with skin	H313	9 (0.1%)	4924 (79.6%)	1076 (17.4%)	18 (0.3%)	159 (2.6%)
Causes severe skin burns and eye damage	H314	615 (9.9%)	5105 (82.5%)	290 (4.7%)	16 (0.3%)	160 (2.6%)
Causes skin irritation	H315	1010 (16.3%)	4714 (76.2%)	287 (4.6%)	15 (0.2%)	160 (2.6%)
Causes mild skin irritation	H316	10 (0.2%)	5706 (92.2%)	294 (4.8%)	16 (0.3%)	160 (2.6%)
May cause an allergic skin reaction	H317	1255 (20.3%)	4317 (69.8%)	428 (6.9%)	26 (0.4%)	160 (2.6%)
Causes serious eye damage	H318	1087 (17.6%)	4574 (73.9%)	352 (5.7%)	14 (0.2%)	159 (2.6%)
Causes serious eye irritation	H319	885 (14.3%)	4762 (77%)	366 (5.9%)	14 (0.2%)	159 (2.6%)
Causes eye irritation	H320	44 (0.7%)	5592 (90.4%)	377 (6.1%)	14 (0.2%)	159 (2.6%)
Fatal if inhaled	H330	119 (1.9%)	3385 (54.7%)	2480 (40.1%)	43 (0.7%)	159 (2.6%)
Toxic if inhaled	H331	188 (3%)	3314 (53.6%)	2482 (40.1%)	43 (0.7%)	159 (2.6%)
Harmful if inhaled	H332	446 (7.2%)	3064 (49.5%)	2474 (40%)	43 (0.7%)	159 (2.6%)
May cause allergy or asthma symptoms or breathing difficulties if inhaled	H334	127 (2.1%)	2054 (33.2%)	3825 (61.8%)	21 (0.3%)	159 (2.6%)
May cause respiratory irritation	H335	377 (6.1%)	4156 (67.2%)	1409 (22.8%)	35 (0.6%)	209 (3.4%)
May cause drowsiness or dizziness	H336	207 (3.3%)	4315 (69.8%)	1415 (22.9%)	35 (0.6%)	214 (3.5%)
May cause genetic defects	H340	143 (2.3%)	4983 (80.6%)	770 (12.4%)	80 (1.3%)	210 (3.4%)
Suspected of causing genetic defects	H341	126 (2%)	5005 (80.9%)	766 (12.4%)	79 (1.3%)	210 (3.4%)
May cause cancer	H350	342 (5.5%)	2260 (36.5%)	3373 (54.5%)	24 (0.4%)	187 (3%)
Suspected of causing cancer	H351	143 (2.3%)	2460 (39.8%)	3372 (54.5%)	24 (0.4%)	187 (3%)
May damage fertility or the unborn child	H360	191 (3.1%)	3854 (62.3%)	1927 (31.2%)	54 (0.9%)	160 (2.6%)
Suspected of damaging fertility or the unborn child	H361	370 (6%)	3677 (59.4%)	1925 (31.1%)	54 (0.9%)	160 (2.6%)
May cause harm to breast-fed children	H362	9 (0.1%)	2132 (34.5%)	3865 (62.5%)	21 (0.3%)	159 (2.6%)
Causes damage to organs	H370	32 (0.5%)	4476 (72.4%)	1414 (22.9%)	35 (0.6%)	229 (3.7%)
May cause damage to organs	H371	24 (0.4%)	4485 (72.5%)	1415 (22.9%)	35 (0.6%)	227 (3.7%)
Causes damage to organs through prolonged or repeated exposure	H372	258 (4.2%)	4466 (72.2%)	1216 (19.7%)	44 (0.7%)	202 (3.3%)
May cause damage to organs through prolonged or repeated exposure	H373	453 (7.3%)	4277 (69.1%)	1212 (19.6%)	44 (0.7%)	200 (3.2%)
Very toxic to aquatic life	H400	805 (13%)	4258 (68.8%)	938 (15.2%)	16 (0.3%)	169 (2.7%)
Toxic to aquatic life	H401	31 (0.5%)	4893 (79.1%)	1078 (17.4%)	16 (0.3%)	168 (2.7%)
Harmful to aquatic life	H402	33 (0.5%)	4888 (79%)	1080 (17.5%)	16 (0.3%)	169 (2.7%)
Very toxic to aquatic life with long-lasting effects	H410	715 (11.6%)	4840 (78.2%)	455 (7.4%)	16 (0.3%)	160 (2.6%)
Toxic to aquatic life with long-lasting effects	H411	870 (14.1%)	4649 (75.2%)	489 (7.9%)	16 (0.3%)	162 (2.6%)
Harmful to aquatic life with long-lasting effects	H412	615 (9.9%)	4904 (79.3%)	489 (7.9%)	16 (0.3%)	162 (2.6%)
May cause long-lasting harmful effects to aquatic life	H413	276 (4.5%)	5240 (84.7%)	492 (8%)	16 (0.3%)	162 (2.6%)
Harms public health and the environment by destroying ozone in the upper atmosphere	H420	3 (0%)	2706 (43.7%)	3272 (52.9%)	8 (0.1%)	197 (3.2%)

**Tab. 5:** Number of substances shared between pairs of toxicological/chemical databases REACH refers only to substances extracted for this publication.

	REACH	ToxRefDB	Tox21	CTD	ChEMBl	PubChem
**REACH**	9,801					
**ToxRefDB**	51	474				
**Tox21**	1,737	375	8,599			
**CTD**	917	230	2,511	13,446		
**ChEMBl**	2,080	339	6,001	5,490	1,715,667	
**PubChem**	4,955	465	8,065	7,729	1,394,860	68,369,258

## Footnotes

1 http://ec.europa.eu/enterprise/sectors/chemicals/reach/index_en.htm

2 http://echa.europa.eu/

3 http://iuclid.eu/

4 https://www.mongodb.org/

5 https://pubchem.ncbi.nlm.nih.gov/score_matrix/score_matrix-help.html

6 https://github.com/gephi/gephi

7 http://actor.epa.gov/toxrefdb/faces/Home.jsp

8 http://epa.gov/ncct/toxcast/data.html

9 http://ctdbase.org/downloads/

10 http://astro.temple.edu/~tua87106/list_fingerprints.pdf

11 https://www.ebi.ac.uk/unichem/analysis/heatoverFullInchi

12 http://ctdbase.org/tools/batchQuery.go
